# Discretization of Non-uniform Rational B-Spline (NURBS) Models for Meshless Isogeometric Analysis

**DOI:** 10.1007/s10915-024-02597-z

**Published:** 2024-07-02

**Authors:** Urban Duh, Varun Shankar, Gregor Kosec

**Affiliations:** 1 Faculty of Mathematics and Physics, University of Ljubljana, Jadranska 19, 1000 Ljubljana, Slovenia; 2 Kahlert School of Computing, University of Utah, Salt Lake City, UT 84112 USA; 3 Parallel and Distributed Systems Laboratory, “Jožef Stefan” Institute, Jamova cesta 39, 1000 Ljubljana, Slovenia

**Keywords:** Meshless, CAD, RBF-FD, Advancing front algorithms, NURBS

## Abstract

We present an algorithm for fast generation of quasi-uniform and variable-spacing nodes on domains whose boundaries are represented as computer-aided design (CAD) models, more specifically non-uniform rational B-splines (NURBS). This new algorithm enables the solution of partial differential equations within the volumes enclosed by these CAD models using (collocation-based) meshless numerical discretizations. Our hierarchical algorithm first generates quasi-uniform node sets directly on the NURBS surfaces representing the domain boundary, then uses the NURBS representation in conjunction with the surface nodes to generate nodes within the volume enclosed by the NURBS surface. We provide evidence for the quality of these node sets by analyzing them in terms of local regularity and separation distances. Finally, we demonstrate that these node sets are well-suited (both in terms of accuracy and numerical stability) for meshless radial basis function generated finite differences discretizations of the Poisson, Navier-Cauchy, and heat equations. Our algorithm constitutes an important step in bridging the field of node generation for meshless discretizations with isogeometric analysis.

## Introduction

A key element of any numerical method for solving partial differential equations (PDE) is discretization of the domain. In traditional numerical methods such as the finite element method (FEM), this discretization is typically performed by partitioning the domain into a mesh, i.e., a finite number of elements that entirely cover it. Despite substantial developments in the field of mesh generation, the process of meshing often remains the most time consuming part of the whole solution procedure while the mesh quality limits the accuracy and stability of the numerical solution [20]. In contrast, meshless methods for PDEs work directly on point clouds; in this context, points are typically referred to as “nodes”. In particular, meshless methods based on radial basis function generated finite difference (RBF-FD) formulas allow for high-order accurate numerical solutions of PDEs on complicated time-varying domains [16, 32] and even manifolds [28]. The generation of suitable nodes is an area of ongoing research, with much work in recent years [9, 11, 31, 35, 39]. In this work, we focus primarily on the generation of nodes suitable for RBF-FD discretizations, although our node generation approach is fully independent of the numerical method used. Node sets may be generated in several different ways. For instance, one could simply generate a mesh using an existing tool and discard the connectivity information [19]. However, such an approach is obviously computationally expensive, not easily generalized to higher dimensions, and in some scenarios even fails to generate node distributions of sufficient quality [31]. Another possible approach is to use randomly-generated nodes [19, 24]; this approach has been used (with some modifications) in areas such as compressive sensing [1] and function approximation in high dimensions [26]. Other approaches include iterative optimization [14, 17, 21], sphere-packing [18], QR factorization [37], and repulsion [11, 39]. It is generally accepted that quasi-uniformly-spaced node sets improve the stability of meshless methods [40]. In this context, methods based on Poisson disk sampling are particularly appealing as they produce quasi-uniformly spaced nodes, scale to arbitrary dimension, are computationally efficient, and can be fully automated [9, 31, 35]. A related consideration is the quality of the domain discretization. In the context of meshes, for instance, it is common to characterize mesh quality using element aspect ratios or determinants of Jacobians [13, 44]. Analogously, the node generation literature commonly characterizes node quality in terms of two measures: the minimal spacing between any pair of nodes (the separation distance), and the maximal empty space without nodes (the fill distance). Once again, in this context, Poisson disk sampling via advancing front methods constitutes the state of the art [9, 35]. More specifically, the DIVG algorithm [35] allows for variable spacing Poisson disk sampling on complicated domains in arbitrary dimension, while its generalization (sDIVG) [9] allows for sampling of arbitrary-dimensional parametric surfaces. DIVG has since been parallelized [7], distributed as a standalone node generator [33], and is also an important component of the open-source meshless project Medusa [36]. Despite these rapid advances in node generation for meshless methods (and in meshless methods themselves), the generation of node sets on domains whose boundaries are specified by computer-aided design (CAD) models is still in its infancy. Consequently, the application of meshless methods in CAD supplied geometries is rare and limited either to smooth geometries [25] or to the use of surface meshes [8, 12, 15]. In contrast, mesh generation and the use of FEM in CAD geometries is a mature and well-understood field [5, 13]. In our experience, current node generation approaches on CAD geometries violate quasi-uniformity near the boundaries and are insufficiently robust or automated for practical use in engineering applications.

In this work, we extend the sDIVG method to the generation of variable spacing node sets on parametric CAD surfaces specified by non-uniform rational B-splines (NURBS). We then utilize the variable-spacing node sets generated by sDIVG in conjunction with the DIVG method to generate node sets in the volume enclosed by the NURBS surface. Our new framework is automated, computationally efficient, scalable to higher dimensions, and generates node sets that retain quasi-uniformity all the way up to the boundary. This framework also inherits the quality guarantees of DIVG and sDIVG, and is consequently well-suited for stable RBF-FD discretizations of PDEs on complicated domain geometries.

The remainder of the paper is organized as follows. The NURBS-DIVG algorithm is presented in Sect. 2 along with analysis of specific components of the algorithm. The quality of generated nodes is discussed in Sect. 3. Its application to the RBF-FD solution of PDEs is shown in Sect. 4. The paper concludes in Sect. 5.

## The NURBS-DIVG Algorithm

CAD surfaces are typically described as a union of multiple, non-overlapping, parametric patches (curves in 2D, surfaces in 3D), positioned so that the transitions between them are either smooth or satisfying some geometric conditions. A popular choice for representing each patch is a NURBS [29], which is the focus of our work. Here, we present a NURBS-DIVG algorithm that has three primary components:

1. First, we extend the sDIVG algorithm [9] (Sect. 2.2) for sampling parametric surfaces to sampling individual NURBS patches and also the union of multiple NURBS patches (Sect. 2.3.2).
2. Next, we deploy the DIVG algorithm [7, 35] in the interior of the domain using the sDIVG generated samples as seed nodes (Sect. 2.1).
3. To ensure that DIVG generates the correct node sets in the interior of the domain whose boundary consists of multiple parametric NURBS patches, we augment sDIVG with a supersampling parameter (Sect. 2.3.3).

In the following subsections, we first briefly present the DIVG algorithm. We then describe the sDIVG algorithm, which generalizes DIVG to parametric surfaces, focusing on sampling a single NURBS surface. We then describe how the sDIVG algorithm is generalized to surfaces consisting of multiple NURBS patches, each of which have their own boundary curves. Finally, we describe the inside check utilized by our algorithm needed to generate nodes within the NURBS patches, and the complications therein.

### The DIVG Algorithm

We now describe the DIVG algorithm for generating node sets within an arbitrary domain. As mentioned previously, this algorithm forms the foundation of the sDIVG and NURBS-DIVG algorithms.

**Fig. 1:**
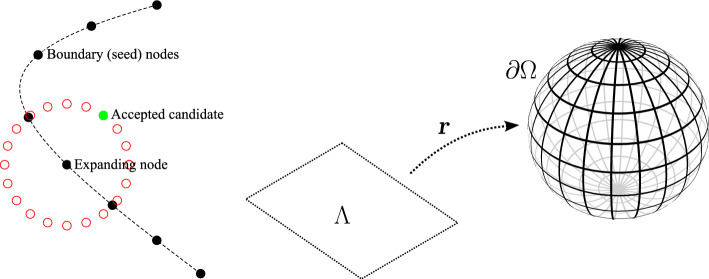
The DIVG expansion scheme (left) and the sDIVG mapping scheme (right)

DIVG is an iterative algorithm that begins with a given set of nodes called “seed nodes”; in our case, these will later be provided by the sDIVG part of the NURBS-DIVG. The seed nodes are placed in an *expansion queue*. In each iteration *i* of the DIVG algorithm, a single node $\varvec{p}_i$ is dequeued and “expanded”. Here, “expansion” means that a set $C_i$ of *n* candidates for new nodes is uniformly generated on a sphere centered at the node $\varvec{p}_i$, with some radius $r_i$ and a random rotation. Here, $r_i$ stands for target nodal spacing and can be thought of as derived from a spacing function *h*, so that $r_i = h(\varvec{p}_i)$ [34, 35]. Of course, the set $C_i$ may contain candidates that lie outside the domain boundary or are too close to an existing node. Such candidates are rejected. The candidates that are not rejected are simply added to the domain and to the expansion queue; this is illustrated in Fig. 1. The iteration continues until the queue is empty. A full description of the DIVG algorithm can be found in [35]. Its parallel variant is described in [7].

### The sDIVG Algorithm

The sDIVG algorithm is a generalization of the DIVG algorithm to parametric surfaces. Unlike DIVG (which fills volumes with node sets), sDIVG instead places nodes on a target parametric surface in such a way that the spacing between nodes on the surface follows a supplied spacing function. While other algorithms typically achieve this through direct Cartesian sampling and elimination [31, 42], the sDIVG algorithm samples the parametric domain corresponding to the surface with an appropriately-transformed version of the supplied spacing function. More concretely, given a domain $\Omega \subset {\mathbb {R}}^d$, sDIVG iteratively samples its boundary $\partial \Omega \subset {\mathbb {R}}^{d}$ by sampling a parametrization $\Lambda$ of its boundary instead. The advantage of this approach over direct Cartesian sampling is obtained from the fact that $\Lambda \subset {\mathbb {R}}^{d-1}$ (or ${\mathbb {S}}^{d-1}$) is a lower-dimensional representation of $\partial \Omega$, leading to an increase in efficiency.

We now briefly describe the spacing function transformation utilized by sDIVG to generate a candidate set for expansion analogous to the one in DIVG. We first define a parametrization ${{\textbf {r}}}: \Lambda \rightarrow \partial \Omega$, i.e., a map from the parametric domain $\Lambda \subset {\mathbb {R}}^{d-1}$ to the manifold $\partial \Omega \subset {\mathbb {R}}^d$; the Jacobian of this function is denoted by $\nabla {{\textbf {r}}}$. As in the DIVG algorithm, let *h* denote the desired spacing function. Now, given a node $\varvec{\lambda }_i \in \Lambda$, we wish to generate a set of *n* candidates for expanding $\varvec{\lambda }_i$, which we write as

1 $$\begin{aligned} C_i = \{{\varvec{\eta }}_{i, j} \in \Lambda ;\ j = 1, \ldots , n\}. \end{aligned}$$

It is important to note that the candidates ${\varvec{\eta }}_{i, j}$ all lie in the parametric domain. Our goal is to determine how far from ${\varvec{\lambda }}_i$ must each candidate lie. From the definition of ${{\textbf {r}}}$ and *h*, the target spacing between the candidate ${\varvec{\eta }}_{i, j}$ and the node being expanded ${\varvec{\lambda }}_i$ is

2 $$\begin{aligned} \Vert {{\textbf {r}}}({\varvec{\eta }}_{i, j}) - {{\textbf {r}}}({\varvec{\lambda }}_i)\Vert = h({{\textbf {r}}}({\varvec{\lambda }}_i)), \end{aligned}$$

for all $j = 1,\ldots ,n$. The candidates ${\varvec{\eta }}_{i, j}$ can be thought of as lying on some manifold around ${\varvec{\lambda }}_i$. This allows us to rewrite ${\varvec{\eta }}_{i, j}$ as

3 $$\begin{aligned} {\varvec{\eta }}_{i, j} = {\varvec{\lambda }}_i + \alpha _{i, j} \vec s_{i, j}, \end{aligned}$$

for some constant $\alpha _{i, j} > 0$ and unit vector $\vec s_{i, j}$. Here, we must appropriately choose the unit vectors $\vec s_{i, j}$ (more on that later) and $\alpha _{i,j}$ must be determined by an appropriate transformation of $h(\textbf{r}({\varvec{\lambda }}_i))$, i.e. the parametric distances $\alpha _{i, j}$ between the candidate and the node being expanded must be obtained by a transformation of the spacing function *h* specified on $\partial \Omega$. We may now use Eq. (3) to Taylor expand ${{\textbf {r}}}({\varvec{\eta }}_{i, j})$ as

4 $$\begin{aligned} {{\textbf {r}}}({\varvec{\eta }}_{i, j}) = {{\textbf {r}}}({\varvec{\lambda }}_i + \alpha _{i, j} \vec s_{i, j}) \approx {{\textbf {r}}}({\varvec{\lambda }}_i) + \alpha _{i, j} \nabla {{\textbf {r}}}({\varvec{\lambda }}_i) \vec s_{i, j}. \end{aligned}$$

We can now use the Taylor expansion in Eq. (4) to approximate the actual spacing between ${\varvec{\lambda }}_i$ and ${\varvec{\eta }}_{i, j}$ in Eq. (2) to obtain the following expression for $h({{\textbf {r}}}({\varvec{\lambda }}_i))$ in terms of $\alpha _{i,j}$:

5 $$\begin{aligned} h({{\textbf {r}}}({\varvec{\lambda }}_i)) \approx \Vert {{\textbf {r}}}({\varvec{\lambda }}_i) + \alpha _{i, j} \nabla {{\textbf {r}}}({\varvec{\lambda }}_i) \vec s_{i, j} - {{\textbf {r}}}({\varvec{\lambda }}_i)\Vert = \alpha _{i, j} \Vert \nabla {{\textbf {r}}}({\varvec{\lambda }}_i) \vec s_{i, j}\Vert . \end{aligned}$$

This in turn allows us to express $\alpha _{i,j}$ as

6 $$\begin{aligned} \alpha _{i, j} = \frac{h({{\textbf {r}}}({\varvec{\lambda }}_i))}{\Vert \nabla {{\textbf {r}}}({\varvec{\lambda }}_i) \vec s_{i, j}\Vert }. \end{aligned}$$

It is important to note here that for such $\alpha _{i, j}$ the target spacing defined in Eq. (2) holds only approximately, i.e., to the first order in the Taylor series expanded in $\alpha _{i, j}$. This is not an issue in practice, since in order to solve PDEs, we typically require node spacings that are small compared to the curvature of the domain boundary $\partial \Omega$. Higher-order approximations can also be computed if needed. We can now use Eq. (6) within Eq. (1) to obtain an explicit expression for the candidate set $C_i$ purely in terms of the spacing function *h* and the parametrization ${{\textbf {r}}}$. Thus, we have

7 $$\begin{aligned} C_i = \left\{ {\varvec{\lambda }}_i + \frac{h({{\textbf {r}}}({\varvec{\lambda }}_i))}{\Vert \nabla {{\textbf {r}}}({\varvec{\lambda }}_i) \vec s_{i, j}\Vert } \vec s_{i, j}; \vec s_{i, j} \in S_i \right\} , \end{aligned}$$

where $S_i$ is set of *n* random unit vectors on a unit ball. All other steps are identical to the DIVG algorithm, albeit in the parametric domain $\Lambda$. The final set of points on $\partial \Omega$ is then obtained by evaluating the function ${{\textbf {r}}}$ at these parametric samples; a schematic of this is shown in Fig. 1 (right). A full description of the sDIVG algorithm and an analysis of its potential weakness can be found in [9].

### NURBS-DIVG

In principle, sDIVG can be used with any map ${{\textbf {r}}}: \Lambda \rightarrow \partial \Omega$. NURBS-DIVG, however, is a specialization of sDIVG to surfaces comprised of NURBS patches (collections of non-overlapping and abutting NURBS). We first describe the use of NURBS for generating the surface representation ${{\textbf {r}}}$, then discuss the generalization to a surface containing multiple patches.

#### An Overview of NURBS Surfaces

To define a NURBS representation, it is useful to first define the corresponding B-spline basis in one-dimension. Given a sequence of nondecreasing real numbers $T = \{t_0, t_1, \dots , t_k\}$ called the *knot vector*, the degree-*p* B-spline basis functions $N_{i, p}(u)$ are defined recursively as [29]

8 $$\begin{aligned} N_{i, 0}(u)&= {\left\{ \begin{array}{ll} 1; &{} t_i \le u < t_{i + 1} \\ 0; &{} \text {otherwise} \end{array}\right. } \end{aligned}$$

9 $$\begin{aligned} N_{i, p}(u)&= \frac{u - t_i}{t_{i + p} - t_i} N_{i, p - 1}(u) + \frac{t_{i + p + 1} - u}{t_{i + p + 1} - t_{i + 1}} N_{i + 1, p - 1}(u) \end{aligned}$$

We can now use these basis function to define a *NURBS curve* in ${\mathbb {R}}^d$. Given a knot vector of the form $T = \{\overbrace{a, \dots , a}^{p + 1 \ \text {times}}, t_{p + 2}, \dots , t_{k - p - 1}, \overbrace{b, \dots , b}^{p + 1 \ \text {times}}\}$, *n* control points ${{\textbf {p}}}_i \in {\mathbb {R}}^d$ and *n* weights $w_i \in {\mathbb {R}}$, the degree-*p* NURBS curve is defined as [29]

10 $$\begin{aligned} {{\textbf {s}}}(u) = \frac{\sum _{i = 0}^{n - 1} N_{i, p}(u) w_i {{\textbf {p}}}_i}{\sum _{i = 0}^{n - 1} N_{i, p}(u) w_i}, \qquad \text {for} \ a \le u \le b. \end{aligned}$$

In practice, it is convenient to evaluate ${{\textbf {s}}}(u) \subset {\mathbb {R}}^d$ as a B-spline curve in ${\mathbb {R}}^{d+1}$, and then project that curve down to ${\mathbb {R}}^d$. For more details, see [29]. We evaluate the B-spline curve in a numerically stable and efficient fashion using the de Boor algorithm [6], which is itself a generalization of the well-known de Casteljau algorithm for Bezier curves [10]. It is also important to note that derivatives of the NURBS curves with respect to *u* are needed for computing surface quantities (tangents and normals, for instance, or curvature). Fortunately, it is well-known that these parametric derivatives are also NURBS curves and can therefore also be evaluated using the de Boor algorithm [29]. We adopt this approach in this work.

Finally, the map ${{\textbf {r}}}$ which represents a surface of co-dimension one in ${\mathbb {R}}^d$ can be represented as a NURBS surface. This surface is obtained in a fairly standard fashion as a tensor-product in knot space, followed by evaluation of the product space through the 1D spline maps. This allows all NURBS surface operations to be computed via the de Boor algorithm applied to each parametric dimension. The sDIVG algorithm can then be used to sample this surface as desired, which in turn allows for node generation in the interior of the domain via DIVG.

**Fig. 2:**
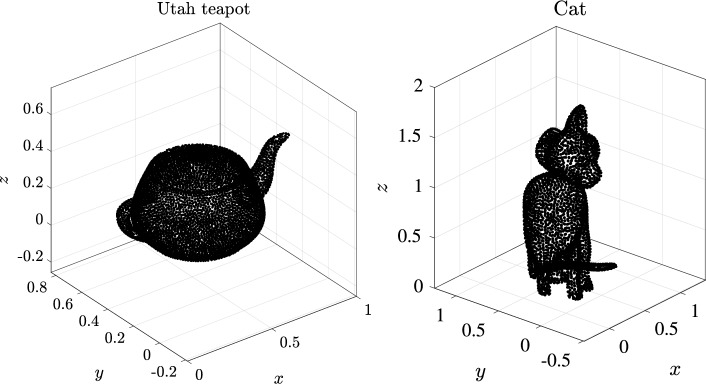
Node sets generated by NURBS-DIVG on the famous Utah Teapot (left) and a CAD model of cat (right) based on [43]. The Utah Teapot model is made of 32 patches and has 7031 boundary nodes; the cat has 211 patches and 3439 boundary nodes

**Fig. 3:**
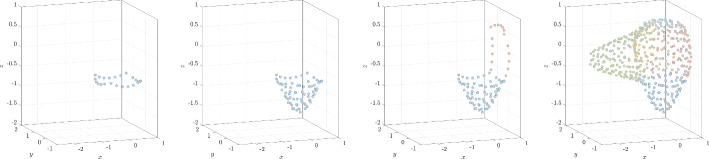
Illustration of positioning nodes on a deformed sphere made of five NURBS patches. In the first step, the boundary of the first patch is filled (first), followed by filling of that patch interior (second). Once the first patch is processed, the boundary of the second patch is discretized (third); this process is repeated until all patches are fully populated with nodes (fourth)

#### Sampling Surfaces Consisting of Multiple NURBS Patches

Practical CAD models typically consist of multiple non-overlapping and abutting NURBS surface patches. A NURBS patch meets another NURBS patch at a NURBS curve (the boundary curves of the respective patches). While degenerate situations can easily arise (such as two NURBS patches intersecting at a single point), we restrict ourselves in this work with patches that intersect in NURBS curves. Some examples of node sets generated by the NURBS-DIVG algorithm on CAD surfaces consisting of multiple NURBS patches are shown in Fig. 2. We now describe the next piece of the NURBS-DIVG algorithm: extending sDIVG to discretize a CAD model that consists of several NURBS patches $\partial \Omega _i$. We proceed as follows:

1. We use sDIVG to populate patch boundaries $\partial (\partial \Omega _i)$ with a set of nodes. Recall that these patch boundaries are NURBS curves.
2. We then use these generated nodes as seed nodes within another sDIVG run, this time to fill the NURBS surface patches $\partial \Omega _i$ enclosed by those patch boundaries.

To populate patch boundaries, the boundary NURBS curve representation obtained from any of the intersecting patches can be used. But, to use nodes from patch boundaries as seed nodes in sDIVG for populating surface patches, the corresponding node from the patch’s parametric domain $\Lambda$ is required. However in general, nodes on intersecting patch boundaries do not necessarily correspond to the same parametric nodes in all the respective parametric domains of intersecting patches. Consequently, the parametric domains from intersecting patches cannot be joined into one “global” parametric domain in a simple and efficient way. While it is possible to determine the map $\Omega \rightarrow \Lambda$ through a nonlinear solve, we found it more efficient to simply populate the patch boundaries twice, once from each of the NURBS representations obtained from intersecting patches. This produces two sets of seed nodes (one corresponding to each patch), but only the set from one of the representations is used in the final discretization (it does not matter which one, since both node sets are of similar quality). The full process is illustrated in Fig. 3.

**Fig. 4:**
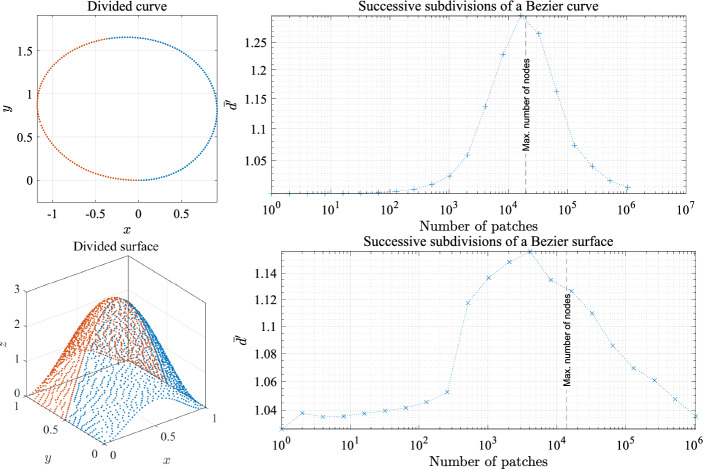
The average normalized distance to *c* nearest neighbors (see Eq. (15) for the precise definition) averaged over the whole domain, $\overline{d'}$, for a discretization of a successively subdivided Bezier curve in 2D and an analogous Bezier surface in 3D. In 2D $c = 2$ and in 3D $c = 3$ are used. Ideally, one strives for ${{\overline{d}}}' = 1$

For a given CAD model consisting of a union of NURBS patches and a desired node spacing *h* (i.e. constant spacing function), it is possible that the smallest dimension of the patch becomes comparable to (or even smaller) than *h*. We now analyze the behavior of sDIVG in this regime. To do so, we construct simple models comprising of Bezier surfaces (NURBS with constant weights); to emulate the existence of multiple patches, we simply subdivide the Bezier surfaces to obtain patches. In the 3D case, each subdivision is performed in a different direction to ensure patches of similar size. The resulting surface, now a union of non-overlapping and abutting NURBS (Bezier) patches, was then discretized with the NURBS-DIVG algorithm using a uniform spacing of $h=10^{-4}$. We then assessed the quality of the resulting node sets on those patches using the normalized local regularity metric ${{\overline{d}}}_i'$ defined in Sect. 3. The models and this metric are shown in Fig. 4. Figure 4 shows that NURBS-DIVG works as expected when *h* is considerably smaller than the patch size. However, once the patch size becomes comparable to the *h*, the NURBS-DIVG algorithm rejects all nodes except those on the boundaries of the patch, as there is not enough space on the patch itself for additional nodes.^1^ In all discussions that follow, we restrict ourselves to the first and most natural regime where *h* is considerably smaller than the patch size.

#### NURBS-DIVG in the Interior of CAD Objects

As our goal is to generate node sets suitable for meshless numerical analysis in volumetric domains, it is vital for the NURBS-DIVG algorithm to be able to generate node sets in the *interior* of volumes whose boundaries are CAD models, in turn defined as a union of NURBS patches. While it may appear that the original DIVG algorithm is already well-suited to this task, we encountered a problem of nodes “escaping” the domain interior when DIVG was applied naively in the CAD setting. We now explain this problem and the NURBS-DIVG solution more clearly.

To discretize the interior of CAD objects, we must accurately determine whether a particular node lies inside or outside the model. The choice of boundary representation can greatly affect the technique used for such an inside/outside test. For instance, if the domain boundary is modeled as an implicit surface (level set) of the form $f(\textbf{x}) = 0$, a node $\textbf{x}_k$ is inside if $f(\textbf{x}_k) < 0$ (up to some tolerance). However, in the case where the domain boundary is modeled as a parametric surface or a collection of parametric patches (as in this work), the analogous approach would be to instead solve a nonlinear system of equations to find the parameter values corresponding to $\textbf{x}_k$ and test if $\textbf{x}_k$ is inside. A simpler approach used in recent work has been to simply find the closest point from the boundary discretization $\textbf{p}$ (with the given spacing *h*) to $\textbf{x}_k$, and use its unit outward normal to decide if $\textbf{x}_k$ is inside the domain. More concretely, if $\textbf{n}$ is the unit outward normal vector at $\textbf{p}$, $\textbf{x}_k$ is inside the domain $\Omega$ when

11 $$\begin{aligned} \textbf{n} \cdot (\textbf{x}_k - \textbf{p}) < 0. \end{aligned}$$

**Fig. 5:**
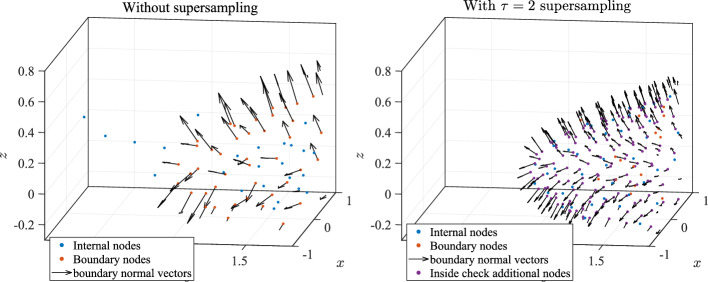
Demonstration of the supersampling approach for the inside/outside test in NURBS-DIVG. The figure on the left shows nodes generated by the naive test used in the DIVG algorithm, with nodes escaping the domain boundary. The figure on the right shows the nodes generated using boundary supersampling in NURBS-DIVG; all non-boundary nodes are enclosed within the volume defined by the boundary

This is the approach used by the DIVG algorithm (and many others). However, in our experience, this does not work well for complex geometries with sharp edges and concavities. For an illustration, see Fig. 5 (left); we see nodes marked as “interior” nodes that are visually outside the convex hull of the boundary nodes.

**Fig. 6:**
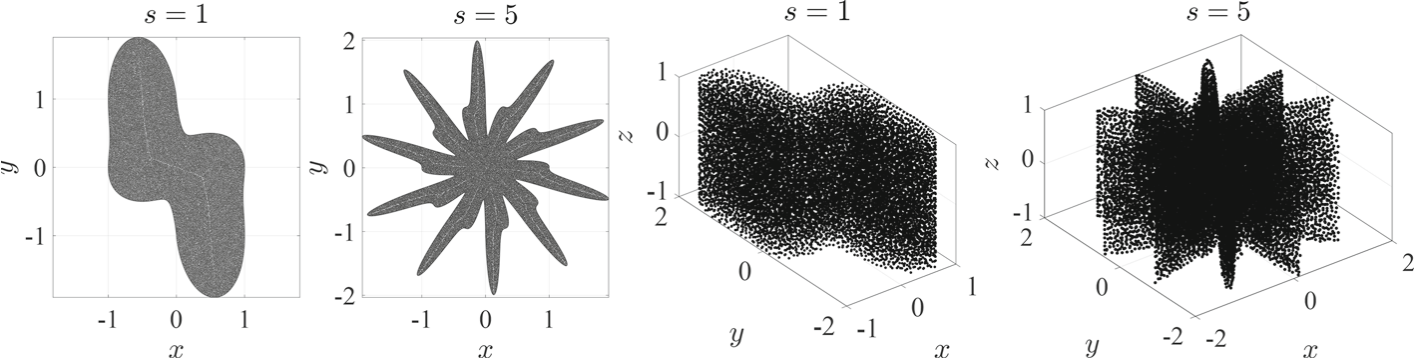
Shapes used to test the supersampling approach in NURBS-DIVG

An investigation revealed that a relatively coarse sampling of a patch near its boundary NURBS curves could result in the closest point $\textbf{p}$ and its normal vector $\textbf{n}$ being a bad approximation of the actual closest point and its normal on the domain $\Omega$, thereby resulting in $\textbf{x}_k$ being erroneously flagged as inside $\Omega$. This problem is especially common on patch boundaries, where normal vectors do not vary smoothly. NURBS-DIVG uses a simple solution: supersampling. More precisely, we use a secondary set of refined boundary nodes only for the inside check with a reduced spacing ${\hat{h}}$ given by

12 $$\begin{aligned} {{\hat{h}}} = h / \tau , \end{aligned}$$

where $\tau > 1$ is a factor that determines the extent of supersampling. Though this potentially requires $\tau$ to be tuned, this solution worked well in our tests with a minimal additional implementation complexity and computational overhead (see execution profiles for Poisson’s equation in Sect. 4). Figure 5 (right) shows the effect of setting $\tau = 2$ in the same domain; nodes no longer “escape” the boundary. While this approach is particularly useful for boundary represented as a collection of NURBS patches, it is likely to be useful in any setting where the boundary has sharp changes in the derivative of the normal vector (or the node spacing *h*). In fact, intuitively, it seems that the greater the derivative (or the bigger the value of *h*), the greater the value of $\tau$ required to prevent nodes escaping. To confirm this intuition, we run a simple test both in 2D and 3D. In 2D, we define a parametric curve

13 $$\begin{aligned} r(t) = |\cos (st)|^{\sin (2st)}, t \in [0, 2\pi ), \end{aligned}$$

where *s* is a parameter controlling the complexity of the curve ($s = 1$ gives 2 legs, $s = 1.5$ gives 3 legs, and so forth). These “legs” create sharp changes in the derivative of the normal (notice *r*(*t*) is not smooth in *t*). In 3D, we simply extrude this curve in the *z* direction to obtain a surface:

14 $$\begin{aligned} {{\textbf {r}}} = (r(t) \cos (t), r(t) \sin (t), z), t \in [0, 2\pi ), z \in [-1, 1]. \end{aligned}$$

**Fig. 7:**
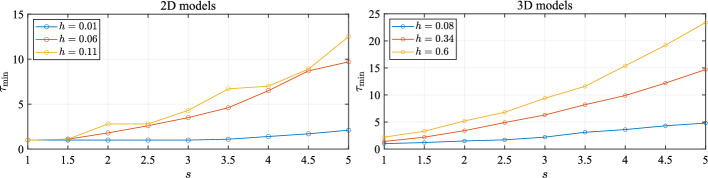
Minimal $\tau$ required to appropriately fill a model

Both test domains are depicted in Fig. 6. We then plot the minimum value of $\tau$ required for a successful inside check as a function of *s*, the parameter that controls the number of legs, and the node spacing *h*. The results are shown in Fig. 7. As expected, increasing the number of legs via *s* necessitates a greater degree of supersampling ($\tau _{min}$ in the plots) in both 2D and 3D. However, if *h* is sufficiently small to begin with, smaller values of $\tau$ appear to suffice. In the tests presented in later sections, we selected *h* to be sufficiently small that $\tau = 2$ sufficed.

## Node Quality

Although node quality in the meshfree context is not as well understood as in mesh based methods, we can analyze local regularity by examining distance distributions to nearest neighbors. For each node ${{\textbf {p}}}_i$ with nearest neighbors ${{\textbf {p}}}_{i, j}, j = 1, \dots c$ we compute

15 $$\begin{aligned} \overline{d_i}&= \frac{1}{c} \sum _{j = 1}^c ||{{\textbf {p}}}_i - {{\textbf {p}}}_{i, j}||, \end{aligned}$$

16 $$\begin{aligned} d_i^\text {min}&= \min _{j=1, \dots , c} ||{{\textbf {p}}}_i - {{\textbf {p}}}_{i, j}||, \end{aligned}$$

17 $$\begin{aligned} d_i^\text {max}&= \max _{j=1, \dots , c} ||{{\textbf {p}}}_i - {{\textbf {p}}}_{i, j}||. \end{aligned}$$

In our analysis, the spacing function *h* is constant over the whole domain, therefore the quantities are normalized as

18 $$\begin{aligned} \overline{d_i'} = \overline{d_i}/h. \end{aligned}$$

For the analysis the following models are selected

**Fig. 8:**
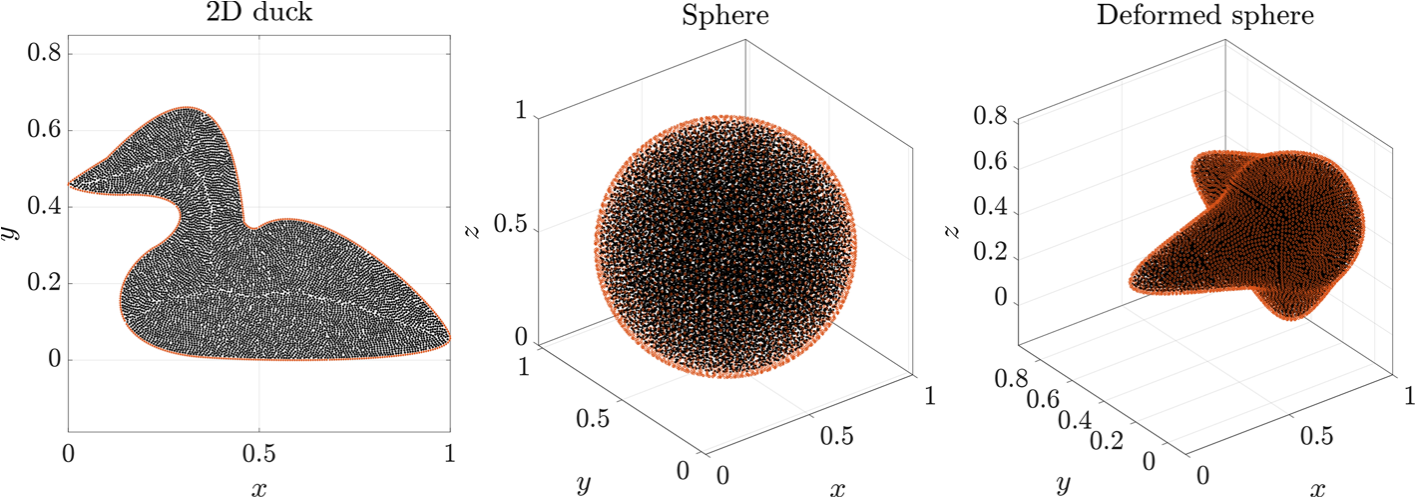
Geometries used in the node quality analysis

- 2D duck with 8 patches,
- sphere with 6 patches,
- deformed sphere with 6 patches,

all depicted in Fig. 8.

**Fig. 9:**
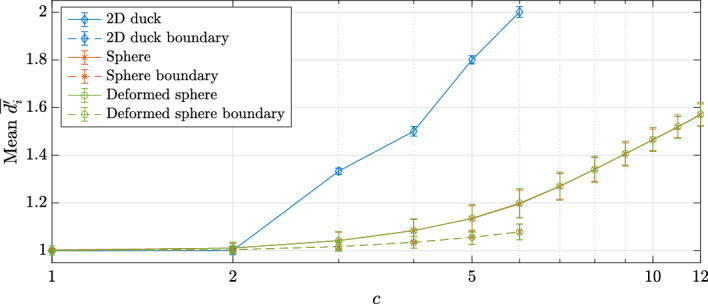
Mean and standard deviation (depicted as error bars) of local regularity distributions $\overline{d_i'}$ computed over the whole domain as a function of the number of nearest neighbors *c* for all three test models

**Fig. 10:**
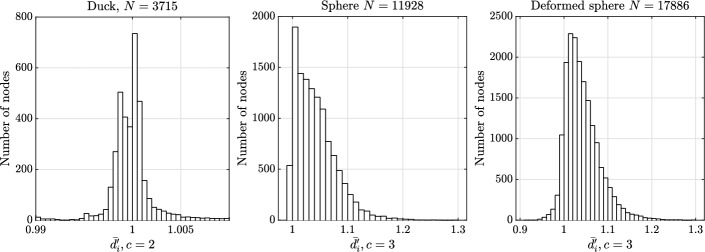
Local regularity distributions for boundary nodes in the case of a constant spacing function

**Fig. 11:**
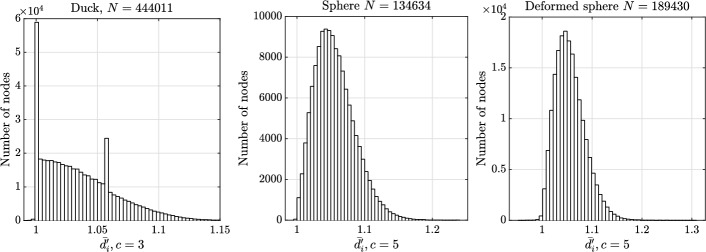
Local regularity distributions for boundary and interior nodes in the case of a constant spacing function

Since the value of ${\overline{d}}_i$ depends on the value of *c*, some reasoning is needed before we analyse our models. Sufficiently far from the boundary, one would ideally like to consider the value of *c* equal to the maximal number of points that can be placed on a unit sphere with mutual distances greater than or equal to 1 (that is 2 in 1D, 6 in 2D and 12 in 3D  [4]). In practice, however, the node distributions are not close to ideal even without the presence of a boundary  [35], which means that considering just the ideal *c* would fail to fairly assess the uniformity of the distribution, especially in the case of a CAD model (where the boundary often plays an important role). In Fig. 9, the means and standard deviations of $\overline{d_i'}$ computed over the whole domain (i.e. the means and averages of distributions later shown in Figs. 10 and 11) are shown as a function of *c* for each considered model. We see that both statistical quantities depend on the model, the dimensionality of the domain, and if we are considering the whole domain or only the boundary. In general, boundaries of a given model are easier to uniformly discretize than the interior, since the boundaries have one dimension less than the interior. This is true despite the fact that sDIVG uses the first order Taylor expansion to determine the appropriate spacing, which results in candidates being generated at spacing only approximately equal to *h*, whereas DIVG makes no such approximation. Furthermore, a simple argument considering only the dimensionalities cannot be sufficient for explaining why the distributions for the 2D duck case are worse than for the 3D boundaries (which is also a 2D object). Here, we must take into account that the duck model has more convex vertices, where, even in the ideal case, one cannot hope to come close to the ideal number of equidistant neighbors for the case of the empty space. Therefore, the distribution of $\overline{d_i'}$ for a large number of nearest neighbors *c* fails to fairly assess the uniformity of nodes. This effect is also later visible in Fig. 11, where a spike just after $\overline{d_i'} = 1.05$ (which can be attributed to said vertices) is visible. The boundary of both 3D objects does not itself have a boundary $\partial (\partial \Omega )$, which means that higher values of *c* give a fairer estimate of node distribution quality there. In following analyses we therefore used $c = 2$ for 1D objects (i.e. domain boundaries in 2D), $c=3$ for 2D objects (domain boundaries in 3D and domain interiors in 2D) and $c = 5$ for 3D objects (domain interiors in 3D).

The distance distributions to nearest neighbors for boundary nodes are presented in Figs. 10, and 11 shows distributions for all nodes. The quantitative statistics are presented in Table 1. It can be seen that the nodes are quite uniformly distributed as all distributions are condensed near 1. In general, the uniformity of boundary node distribution is on par with the distribution of interior nodes.

In the 2D duck case, the distribution of boundary nodes visually seems much better than in the 3D cases. This is a consequence of the candidate generation procedure, which is more optimal when the parametric domains are 1D. Another feature characteristic for 1D parametric domains is that outliers with distance to nearest neighbors slightly less than 2*h* are not uncommon. This happens at nodes where the advancing fronts of the sDIVG algorithm meet and is rarely a problem in practice. For these reasons, the standard deviation for duck case shown in Table 1 is of the same order of magnitude as the 3D cases. If we remove the 10 most extreme outliers, the standard deviation reduces by an order of magnitude. See [9] for a deeper analysis and possible solutions.

In the 3D cases, the distribution of nodes for the deformed sphere is slightly worse, which can be attributed to a greater complexity of the model. Nevertheless, the quality of generated nodes is of the same order as the nodes generated by pure DIVG [35] and sDIVG [9].

**Table 1:** Statistics of local regularity distributions shown in Figs. 10 and 11

		${\text {mean}}{\bar{d}}'_i$	${\text {std}}{\bar{d}}'_i$	${\text {mean}}\left( \left( d_i^{\text {max}}\right) ' - \left( d_i^{\text {min}}\right) '\right)$
Boundary nodes	Duck	1.0007	0.017	0.0022
	Sphere	1.04	0.036	0.10
	Deformed sphere	1.04	0.039	0.10
Boundary and interior nodes	Duck	1.036	0.030	0.101
	Sphere	1.055	0.029	0.103
	Deformed sphere	1.056	0.030	0.140

Additionally, there are two quantities often considered as node quality measures, i.e., minimal distance between nodes (also referred to as separation distance) and fill distance (also referred to as the maximal empty sphere radius) within the domain [14, 40]. The minimal distance is defined for set of nodes $\Xi = \{x_1, \dots , x_N\} \subset \Omega$ as

19 $$\begin{aligned} r_{\text {min}, \Xi } = \frac{1}{2} \min _{i \ne j} ||{{\textbf {x}}}_i - {{\textbf {x}}}_j|| \end{aligned}$$

and fill distance as

20 $$\begin{aligned} r_{\text {max}, \Xi } = \sup _{{{\textbf {x}}} \in \Omega } \min _i ||{{\textbf {x}}} - {{\textbf {x}}}_i||. \end{aligned}$$

Quantity $r_{\text {min}, \Xi }$ is determined by finding the nearest neighbor for all nodes using a spatial search structure, such as a *k*-d tree. A $r_{\text {max}, \Xi }$ is estimated numerically by sampling $\Omega$ with higher node density and searching for the closest node among $\Xi$.

**Fig. 12:**
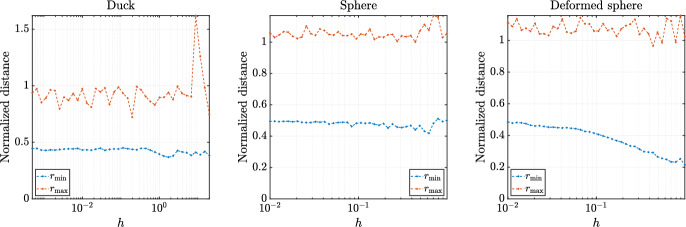
Minimal and fill distance on the boundary with respect to different constant values of the spacing function *h*

**Fig. 13:**
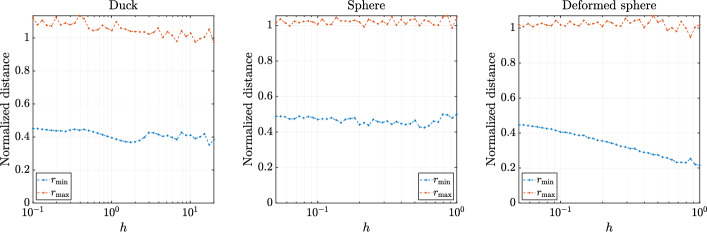
Minimal and fill distance on the whole domain with respect to different constant values of the spacing function *h*

The behaviour of the normalized fill distance and separation distance for all three cases with respect to target nodal distance *h* is presented in Figs. 12 and 13. In all cases, $r_\text {max}$ is relatively stable near an acceptable value of 1 and $r_\text {min}$ approaches the optimal value of 0.5 with decreasing *h*. This behaviour is consistent with previous results and the analytical bound for $r_\text {min}$ for sDIVG [9, 35].

## Solving PDEs on CAD Geometry

In this section, we focus on solving PDEs on domains discretized with scattered nodes ${{\textbf {x}}}_i$ using the new NURBS-DIVG algorithm. In each node ${{\textbf {x}}}_i$, the partial differential operator ${\mathcal {L}}$ is approximated using a set of *n* nearest nodes, commonly referred to as support domain or stencil, as

21 $$\begin{aligned} {\mathcal {L}}u({{\textbf {x}}}_i) \approx \sum _{j=1}^n w_j u({{\textbf {x}}}_{i,j}), \end{aligned}$$

where index *j* runs over the stencil nodes of a node $\textbf{x}_i$, ${{\textbf {w}}}$ are weights still to be determined and $u(\textbf{x}_{i,j})$ stands for the function *u* evaluated at the *j*-th stencil node of the node ${{\textbf {x}}}_i$. The weights are determined by solving a linear system resulting from enforcing the equality of the Eq. (21) for the set of approximation basis functions. In our case, the basis consists of polyharmonic splines (PHS)  [3] that are centered at the stencil nodes, augmented with polynomials up to order *m*. Such a setup corresponds to a meshless method commonly referred to as the Radial basis function-generated finite differences (RBF-FD) [2, 3, 16, 30, 38]. For the purposes of this work, we used the RBF-FD implementation discussed in [22, 36] with augmentation up to order $m \in \{2, 4, 6\}$ on $$n = 4 \left( {\begin{array}{c}m + 2\\ 2\end{array}}\right) $$ closest nodes in 2D and $$n = 4 \left( {\begin{array}{c}m + 3\\ 3\end{array}}\right) $$ in 3D to obtain the mesh-free approximations of the differential operators involved.

### Poisson’s Equation

**Fig. 14:**
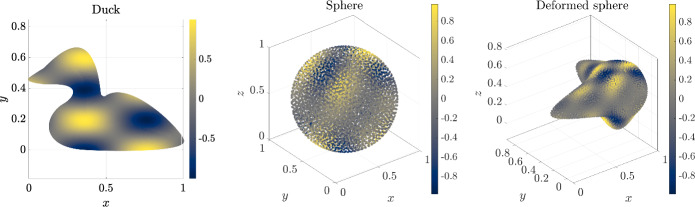
Solution of Poisson’s equation on all three test geometries

First, we solve the Poisson’s equation

22 $$\begin{aligned} \nabla ^2 u = f \end{aligned}$$

with a known closed-form solution

23 $$\begin{aligned} u_a(x, y)&= \sin \left( \frac{\pi }{100}x\right) \cos \left( \frac{2\pi }{100} y\right) , \quad \text {in 2D}, \end{aligned}$$

24 $$\begin{aligned} u_a(x, y, z)&= \sin (\pi x) \cos (2 \pi y) \sin (0.5 \pi z), \quad \text {in 3D,} \end{aligned}$$

using mixed Neumann-Dirichlet boundary conditions

25 $$\begin{aligned} u&= u_a, \quad \text {on } \Gamma _d, \end{aligned}$$

26 $$\begin{aligned} \frac{\partial {u}}{\partial {{\textbf {n}}}}&= \frac{\partial {u_a}}{\partial {{\textbf {n}}}}, \quad \text {on } \Gamma _n, \end{aligned}$$

where $\Gamma _d$ and $\Gamma _n$ stand for Dirichlet and Neumann boundaries. In all cases, the domain boundary is divided into two halves, where we apply a Dirichlet boundary condition to one half and a Neumann boundary condition to another. The numerical solution of the problem is presented in Fig. 14.

Once the numerical solution ${\hat{u}}$ is obtained, we observe the convergence behaviour of the solution through error norms defined as

27 $$\begin{aligned} e_1&= \frac{\Vert {\hat{u}} - u_a\Vert _1}{\Vert u_a\Vert _1}, \quad \Vert u_a\Vert _1 = \frac{1}{N} \sum _{i=1}^N |u_a^i|, \end{aligned}$$

28 $$\begin{aligned} e_2&= \frac{\Vert {\hat{u}} - u_a\Vert _2}{\Vert u_a\Vert _2}, \quad \Vert u_a\Vert _2 = \sqrt{\frac{1}{N} \sum _{i=1}^N |u_a^i|^2}, \end{aligned}$$

29 $$\begin{aligned} e_{\infty }&= \frac{\Vert {\hat{u}} - u_a\Vert _\infty }{\Vert u_a\Vert _\infty }, \quad \Vert u_a\Vert _\infty = \max _{i=1, \ldots , N}|u_a^i|. \end{aligned}$$

**Fig. 15:**
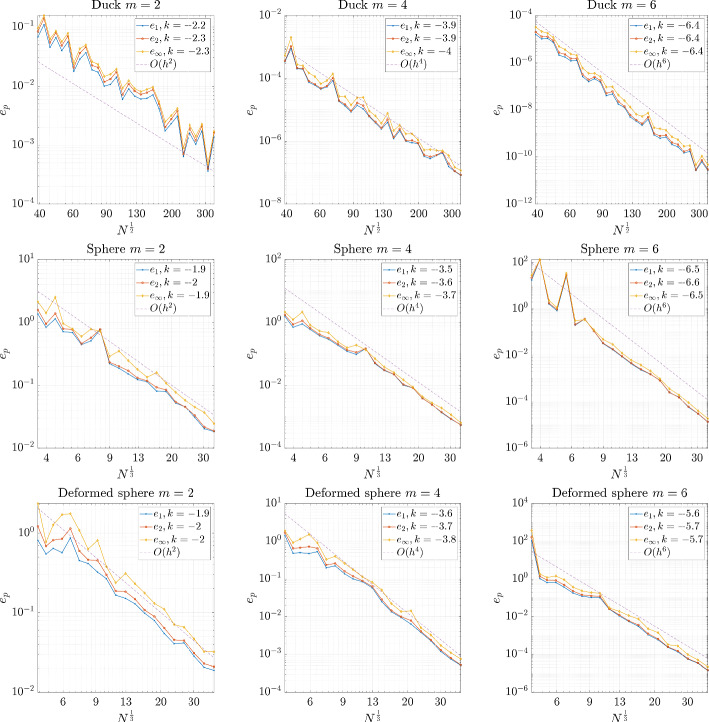
Error in the solution to Poisson’s equation with respect to the number of nodes

In Fig. 15 we can see that for all three geometries the solution converges with the expected order of accuracy according to the order of augmenting monomials.

**Fig. 16:**
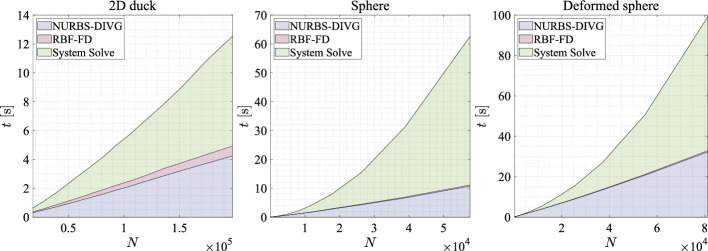
Execution times broken down to separate solution procedure modules

Next, we assess the execution time of solving Poisson’s equation with second order monomial augmentation. In Fig. 16 the execution times for all three geometries are broken down to core modules of the solution procedure. We measure execution time for generation of nodes using proposed NURBS-DIVG algorithm.^2^ The generation of stencils and the computation of stencil weights are measured together as the RBF-FD part of the solution procedure. Separately, we also measure the cost of sparse matrix assembly (which is negligible [7]) and the solution of the corresponding linear system; with an increasing number of nodes, this solve ultimately dominates the execution time [7]. In the 2D duck case, the computational times of solving the system and filling the domain are of the same order as the number of nodes is still relatively small. However, in both 3D cases we can clearly see that the cost of solving the linear system scales super-linearly and soon dominates the overall computational cost. The RBF-FD part (as expected) scales almost linearly (neglecting the ${\mathcal {O}}(N\log {N})$ resulting from *k*-d tree in stencil selection).

### Linear Elasticity—Navier-Cauchy Equation

In the previous section, we established confidence in the presented solution procedure by obtaining expected convergence rates in solving Poisson’s equation on three different geometries in 2D and 3D. In this section we apply NURBS-DIVG to a more realistic case from linear elasticity, governed by the Navier-Cauchy equation

30 $$\begin{aligned} \frac{E}{2\left( \nu + 1 \right) }\left( \nabla ^2 {{\textbf {u}}} + \frac{1}{1 - 2 \nu }\nabla \left( {\nabla \cdot {{\textbf {u}}}} \right) \right) = 0 \end{aligned}$$

where $\varvec{u}$ stands for the displacement vector, and Young’s modulus $E = {72.1\,\cdot \,10^{9}}\,\text {Pa}$ and Poisson’s ratio $\nu = 0.33$ define material properties. The displacement and the stress tensor ($\sigma$) are related via Hooke’s law

31 $$\begin{aligned} \sigma = \frac{E}{\nu + 1} \left( \frac{1}{1 - 2 \nu } \text {tr}(\varepsilon ) I + \varepsilon \right) , \quad \varepsilon = \frac{\nabla \textbf{u} + (\nabla {{\textbf {u}}})^{\textsf{T}}}{2}, \end{aligned}$$

with $\varepsilon$ and *I* standing for strain and identity tensors. We observe a 3D gear object that is subjected to an external torque resulting in a tangential traction $t_0 = {1\,\cdot \,10^3}\,\text {Pa}$ on axis, while the gear teeth are blocked, i.e. the displacement is zero ${{\textbf {u}}} = {0}\,\text {m}$. The top and bottom surfaces are free, i.e. traction free boundary conditions apply. In summary

32 $$\begin{aligned} \textbf{u}&= {0}\,\text {m}, \quad \text {on } \Gamma _{\text {teeth}}, \end{aligned}$$

33 $$\begin{aligned} \sigma \cdot {{\textbf {n}}}&= {0}\,\text {Pa}, \quad \text {on } \Gamma _{\text {free}}, \end{aligned}$$

34 $$\begin{aligned} \sigma \cdot {{\textbf {t}}}&= t_0 {{\textbf {t}}}, \quad \text {on } \Gamma _{\text {axis}}. \end{aligned}$$

**Fig. 17:**
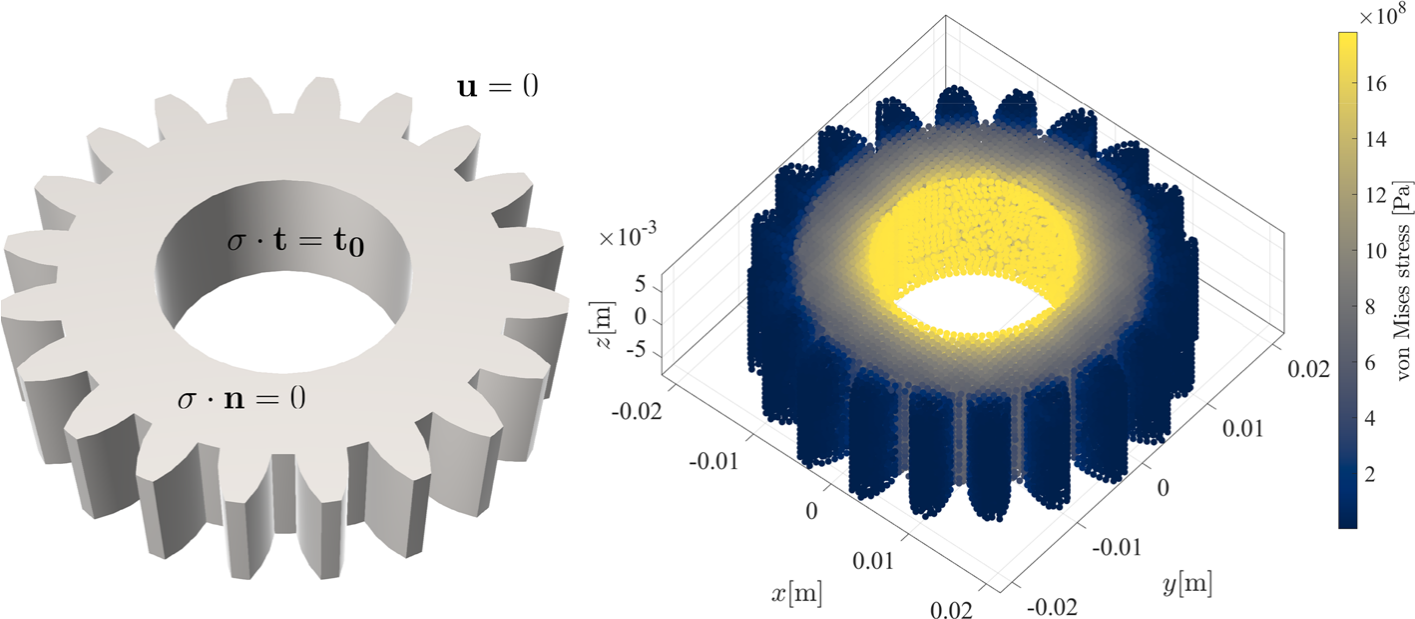
Scheme of the linear elasticity example (left) accompanied with the RBF-FD solution in terms of von Misses stress (right). The gear model is made of 84 patches

**Fig. 18:**
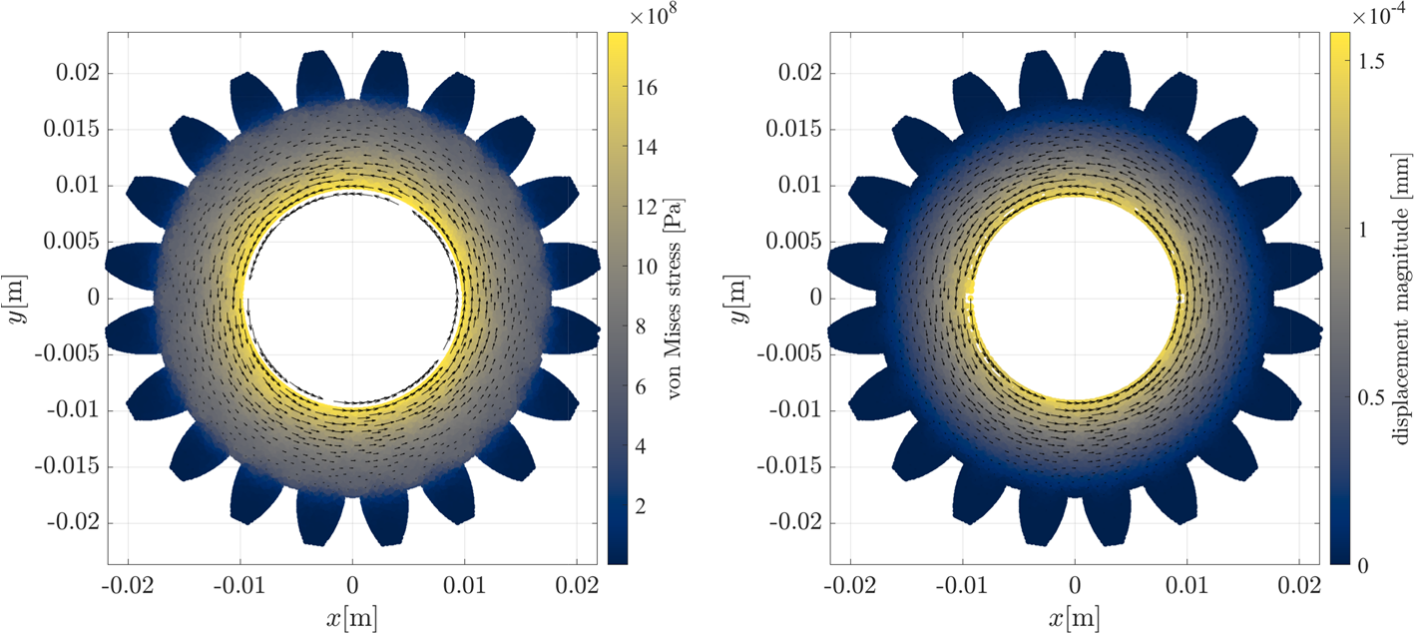
The von Mises stress (left) and the displacement magnitude (right) at $z=0$ cross section accompanied with a quiver plot of the displacement field

The case is schematically presented in Fig. 17 together with von Mises stress scatter plot. The stress is highest near the axis where the force is applied, and gradually fades towards blocked gear teeth. Displacement and von Mises stress are further demonstrated in Fig. 18 at $z = {0}\,\text {m}$ cross section, where we see how the gear is deformed due to the applied force. All results were computed using 41210 scattered nodes generated by NURBS-DIVG.

### Transient Heat Transport

The last example is focused on the transient heat equation

35 $$\begin{aligned} \frac{\partial T}{\partial t} = \lambda \nabla ^2 T + q, \end{aligned}$$

where *T* stands for temperature, $\lambda$ for thermal conductivity, and *q* for the heat source. The goal is to solve heat transport within the duck model subject to the Robin boundary condition

36 $$\begin{aligned} \frac{\partial T}{\partial t} + T = 0 \end{aligned}$$

and a heat source within the domain

37 $$\begin{aligned} q = 5 e^{10||{{\textbf {x}}} - {{\textbf {x}}}_0||} \end{aligned}$$

with ${{\textbf {x}}}_0 = (0,0,0.2)$, the initial temperature set to 0 throughout the domain, and $\lambda = 2$. Time marching is performed via implicit stepping

38 $$\begin{aligned} \frac{T_2 - T_1}{\Delta t} = \lambda \nabla ^2 T_2 + q, \end{aligned}$$

where $T_1$ and $T_2$ stand for the temperature in the current and the next time step respectively and $\Delta t$ represents the time step. The spatial discretization of the Laplace operator is done using RBF-FD with $m=2$. We used a time step of $\Delta t = 3 \cdot 10^{-4}$ and 3000 iterations to reach the steady state using the criterion $T_2 - T_1 < 3\cdot 10^{-6}$) at $t=0.9$.

**Fig. 19:**
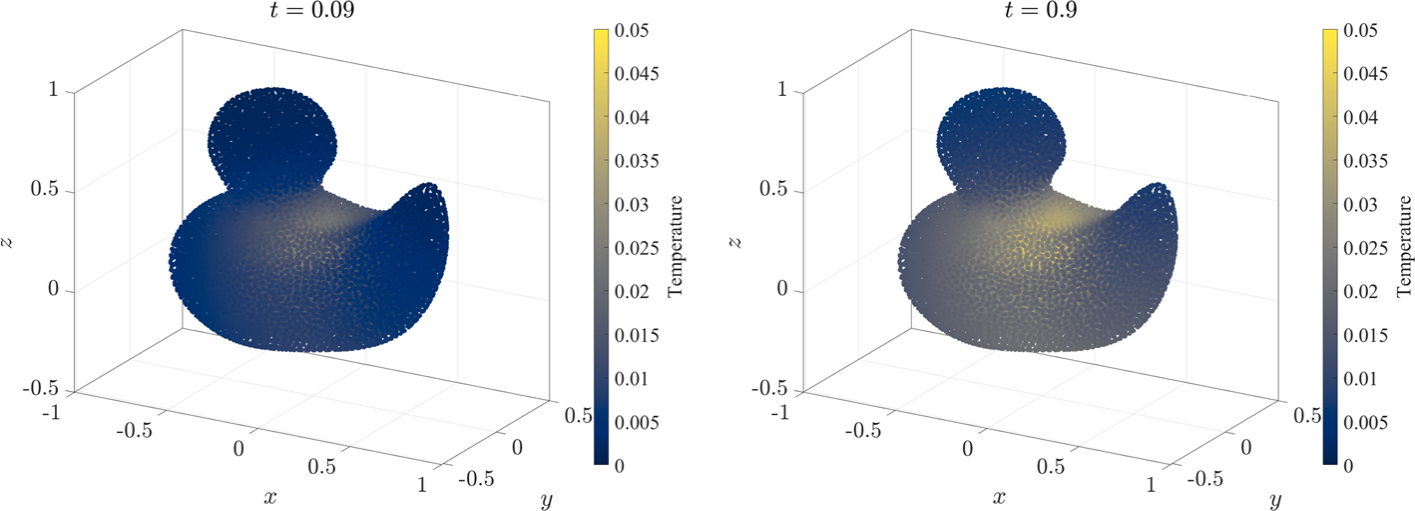
Heat transport within a 3D duck. The model is based on [41] and consists of only 1 patch

**Fig. 20:**
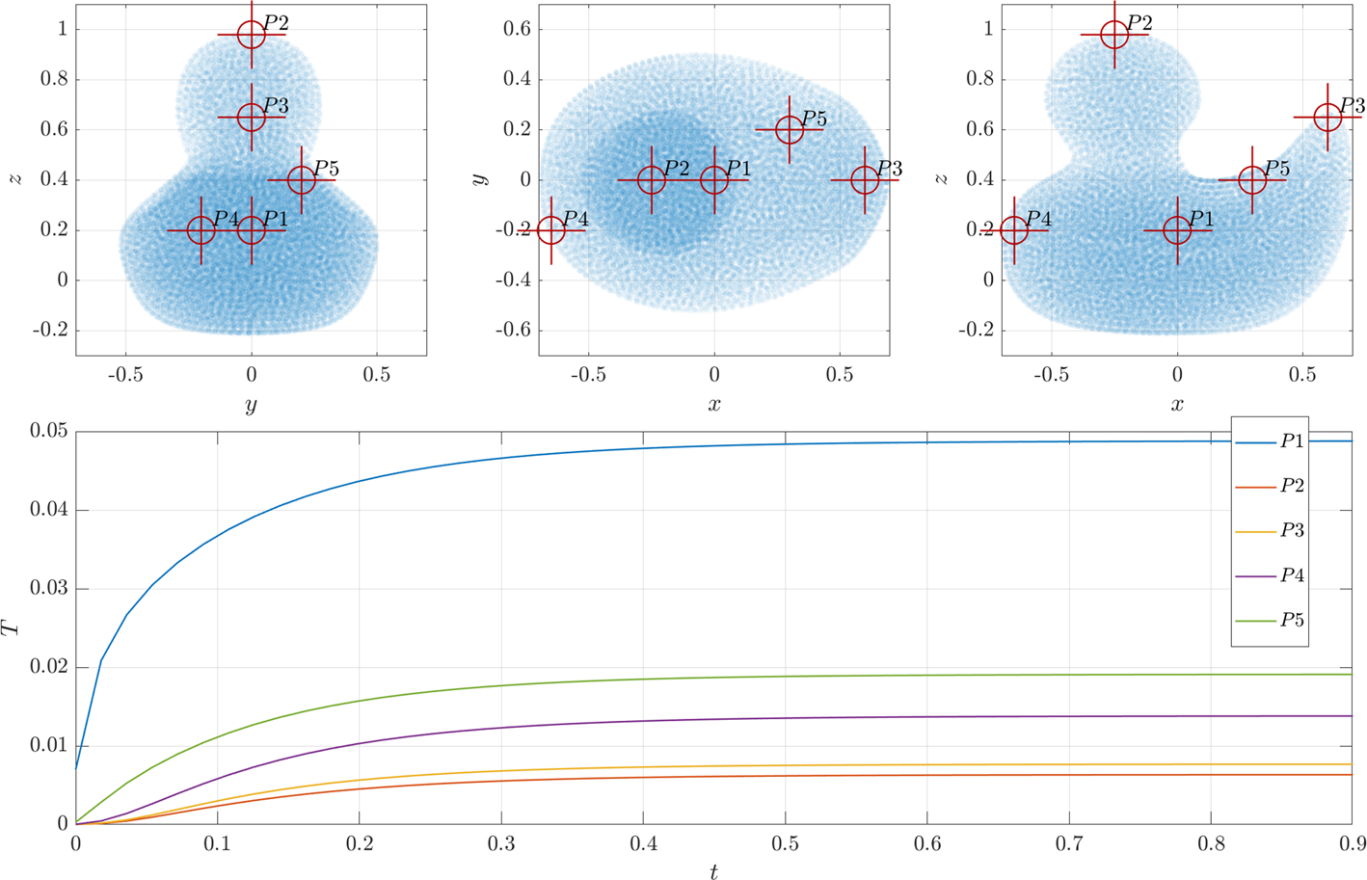
Time evolution of the temperature at five control points

Figure 19 shows the temperature scatter plot computed with RBF-FD on 21956 nodes generated with the proposed NURBS-DIVG at two different times (first at the beginning of the simulation and second at the steady state). In Fig. 20, the time evolution of the temperature at five control points $P1-5$ is shown. Control point *P*1 is located at the heat source, $P2-4$ at the most distant points from the source, and *P*5 asymmetric with respect to the y-axis. As one would expect, at the source the temperature rises immediately after the beginning of the simulation and also reaches the highest value, while the rise is a bit delayed and lower at the distant points that are closer to the boundary where the heat exchange with surroundings takes place. Once the heat exchange with the surroundings matches the heat generation at the source, the system reaches steady-state.

## Conclusions

In this paper, we presented a meshless algorithm, NURBS-DIVG, for generating quasi-uniform nodes on domains whose boundaries are defined by CAD models consisting of multiple NURBS patches. The NURBS-DIVG algorithm is able to deal with complex geometries with sharp edges and concavities, supports refinement, and can be generalized to higher dimensions. We also demonstrated that node layouts generated with NURBS-DIVG are of sufficiently high quality for meshless discretizations, first by directly assessing the quality of these node sets, then by using RBF-FD to solve the Poisson equation with mixed Dirichlet-Neumann boundary conditions on different domains to high-order accuracy. Finally, we demonstrated NURBS-DIVG in conjunction with RBF-FD in tackling two more challenging test cases: first, the stress analysis of a gear subjected to an external force governed by the Navier-Cauchy equation; and second, a time-dependent heat transport problem inside a duck. This work advances the state of the art in fully-autonomous, meshless, isogeometric analysis. All algorithms presented in this work are implemented in C++ and included in our in-house open-source meshfree library *Medusa* [22, 36], see the *Medusa wiki* [23] for usage examples. The interface to all CAD files was implemented via Open Cascade [27].

## Data Availability

The datasets generated during and/or analysed during the current study are available from the corresponding author on reasonable request. For some practical examples see [23].

## Footnotes

1 Of course, the figures also show that in the case where *h* is bigger than the average patch size, NURBS-DIVG works well again, since this scenario is analogous to choosing which patches to place nodes in. However, this scenario is not of practical interest.

2 For a more in-depth analysis of computational complexity, see the DIVG [35] and sDIVG [9] papers.
